# CHD1L promotes EOC cell invasiveness and metastasis via the regulation of METAP2

**DOI:** 10.7150/ijms.48615

**Published:** 2020-08-29

**Authors:** Wei-Peng He, Yun-Yun Guo, Gui-Ping Yang, Hui-Ling Lai, Ting-Ting Sun, Zu -Wei Zhang, Ling-Long Ouyang, Yu Zheng, Li-Ming Tian, Xiao-Hui Li, Ze-Shan You, Dan Xie, Guo-Fen Yang

**Affiliations:** 1 Department of Gynecology, the First Affiliated Hospital, Sun Yat-Sen University, No. 58, Zhongshan Road II, 510080 Guangzhou, China.; 2 State Key Laboratory of Oncology in South China, Cancer Center, Sun Yat-Sen University, No. 651, Dongfeng Road East, 510060 Guangzhou, China.

**Keywords:** Ovarian cancer, CHD1L, invasion, metastasis, METAP2

## Abstract

Chromodomain helicase DNA binding protein 1-like (CHD1L) gene has been proposed to play an oncogenic role in human hepatocellular carcinoma. Previously we reported that CHD1L overexpression is significantly associated with the metastasis proceeding of epithelial ovarian cancer (EOC), and may predict a poor prognosis in EOC patients. However, the potential oncogenic mechanisms by which CHD1L acts in EOC remain unclear. To elucidate the oncogenic function of CHD1L, we carried out a series of *in vitro* assays, with effects of CHD1L ectogenic overexpression and silencing being determined in EOC cell lines (HO8910, A2780 and ES2). Real-time PCR and Western blotting analyses were used to identify potential downstream targets of CHD1L in the process of EOC invasion and metastasis. In ovarian carcinoma HO8910 cell lines, ectopic overexpression of CHD1L substantially induced the invasive and metastasis ability of the cancer cells *in vitro*. In contrast, knockdown of CHD1L using shRNA inhibited cell invasion *in vitro* in ovarian carcinoma A2780 and ES2 cell lines. We also demonstrated that methionyl aminopeptidase 2 (METAP2) was a downstream target of CHD1L in EOC, and we found a significant, positive correlation between the expression of CHD1L and METAP2 in EOC tissues (P<0.05). Our findings indicate that CHD1L plays a potential role in the inducement of EOC cancer cell invasion and/or metastasis via the regulation of METAP2 expression and suggests that CHD1L inhibition may provide a potential target for therapeutic intervention in human EOC.

## Introduction

Ovarian cancer is a leading type of cancer among cancers afflicting the female reproductive system and it is the leading cause of death among all gynecological malignancies 1. The most common pathological type of ovarian cancer is epithelial ovarian cancer (EOC), and it accounts for 95% of all cases of ovarian cancer. Because EOC is not often accompanied by noticeable symptoms, most patients are not diagnosed until an advanced stage (FIGO III or IV), resulting in a devastating overall 5-year survival rate of around 30% 2. Due to the limiting efficacy of chemotherapy, satisfactory tumor cell subtraction is still the most effective treatment for EOC 3, with the greatest challenge arising from extensive metastasis of the pelvic cavity. In addition, metastasis to distal regions of the body outside of the reproductive system is still considered a major factor in the recurrence and possible death in EOC patients 4.

It has been well established that the occurrence and development of EOC is a complex process involving multiple genetic changes, all with multiple steps 5. Therefore, it is a pressing problem to discover and identify the molecular mechanisms of the genes involved in pelvic and peritoneal metastasis in order to improve EOC treatment efficiency and prognosis.

CHD1L (chromodomain helicase DNA binding protein 1-like), also called ALC1 (amplified in liver cancer 1), is a putative oncogene possibly involved in EOC located at chromosome 1q21. The mRNA of CHD1L is 3036 bp long, resulting in a CHD1L protein 897 amino acids in length. The CHD1L protein belongs to SNF2-like family and contains a SNF2-N domain and a HELICs (helicase superfamily c-terminal domain) structure. Most members of the SNF2-like family participate in various nuclear activities, such as transcriptional activation or repression, DNA repair, and recombination. CHD1L is named so due to it being 59% homologous to CHD1 (chromodomain helicase DNA binding protein 1) which is responsible for modifying chromatin structure 6-8. Previous work has shown that CHD1L is overexpressed in a variety of human malignancies, including colorectal cancer, breast cancer, gastric cancer, nasopharyngeal cancer, lung cancer, pancreatic cancer, esophageal carcinoma and ovarian cancer, and the degree of CHD1L overexpression is correlated with tumor progression and poor prognosis 9-16. Previously we reported CHD1L overexpression is significantly correlated with the metastasis proceeding of EOC, and may also be an accurate predictor of poor prognosis in EOC patients 16, suggesting that CHD1L may participate in tumor metastasis. Although CHD1L expression has been extensively characterized in human cancers, the molecular mechanisms underlying aberrant CHD1L expression induced in human malignancies remain unknown.

In order to illuminate the possible roles of CHD1L in the development of EOC, here we investigate the tumorigenicity of CHD1L and the potential molecular mechanisms involved in the invasion and metastasis during EOC, using EOC cell lines. Our results might shed light into the molecular mechanisms of CHD1L involvement in the development, progression and metastasis of EOC.

## Results

### Expression of CHD1L in a large cohort of ovarian tissues and its correlation with the clinicopathologic features and survival of EOC patients

**Figure 1:**
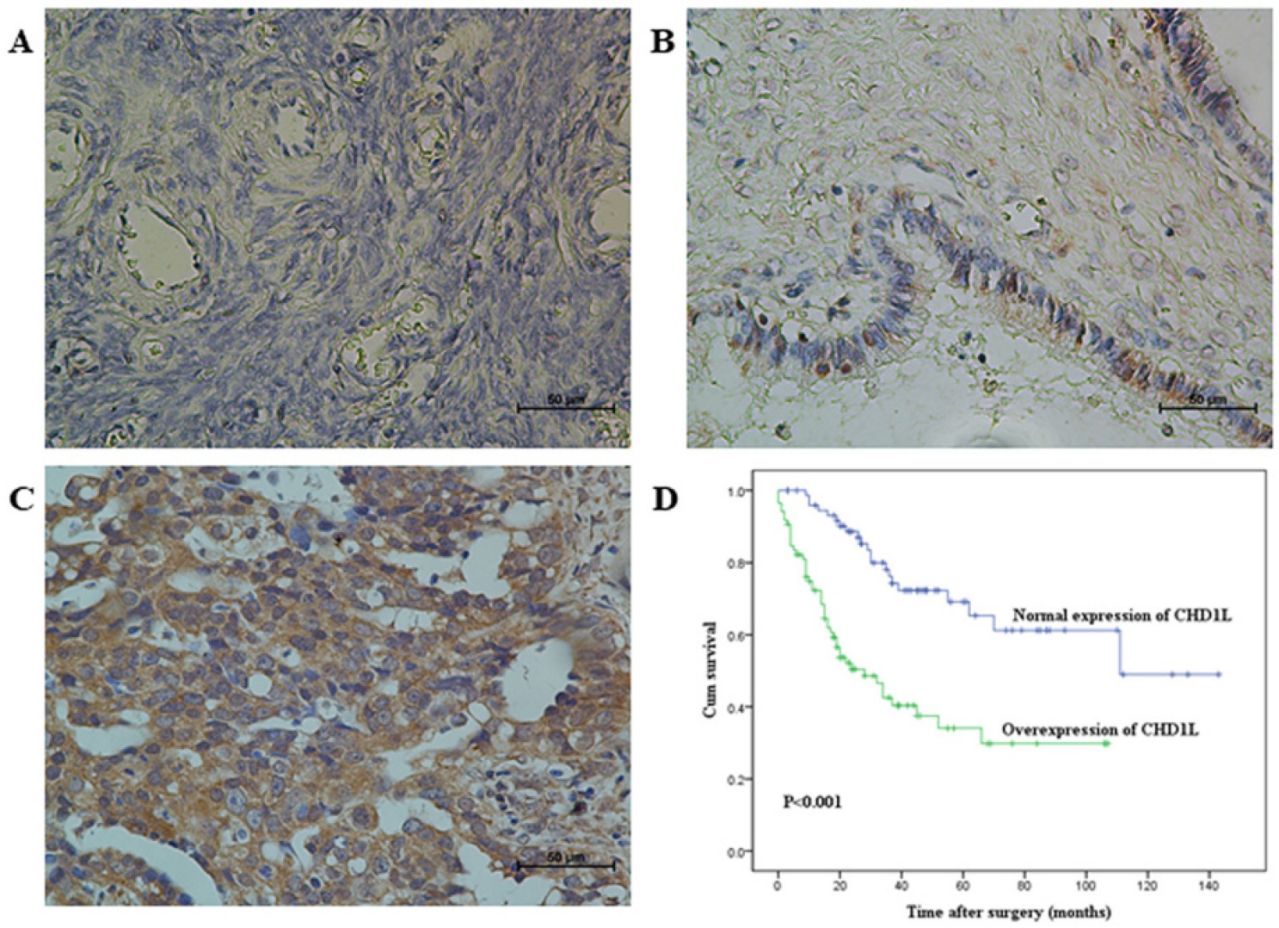
** Immunohistochemical staining of CHD1L in human ovarian tissues and Kaplan-Meier survival analysis according to CHD1L expression in 160 EOC patients (log-rank test). A.** Normal expression of CHD1L was observed in a normal surface epithelium of ovary (400×). **B.** An ovarian cystadenoma showed low expression of CHD1L, in which about 20% of tumor cells showed moderate positive staining of CHD1L (400×). **C.** Overexpression of CHD1L was detected in an ovarian carcinoma (case 93), in which more than 90% of carcinoma cells showed strong positive staining of CHD1L (400×). **D.** Probability of survival of patients: overexpression of CHD1L, n=85; normal expression of CHD1L, n=75 (*P*<0.001).

Using a large cohort of clinical EOC tissue, borderline tumors, cystadenomas tissue, and normal ovarian tissue for control, the expression dynamics of CHD1L were evaluated using immunohistochemistry (IHC). Using previously described protein expression criteria 16, we found that overexpression of CHD1L was observed in 53%, 13% and 6% of specimens of EOC, borderline tumors, and cystadenomas, respectively, with expectedly no overexpression observed in any normal ovarian tissue sample (*P*<0.01, **Table 1, Fig. 1A-C**). Upon further analysis, we found that overexpression of CHD1L was significantly, positively correlated with histological type and advanced pT/pN/pM status, FIGO stage of ovarian carcinomas (*P*<0.05, **Table 2**), and poor survival rates in EOC patients (*P*<0.001, **Fig. 1D**).

### Ectogenic overexpression of CHD1L transfected with plasmid promotes EOC cell invasion and metastasis * in vitro*

**Figure 2:**
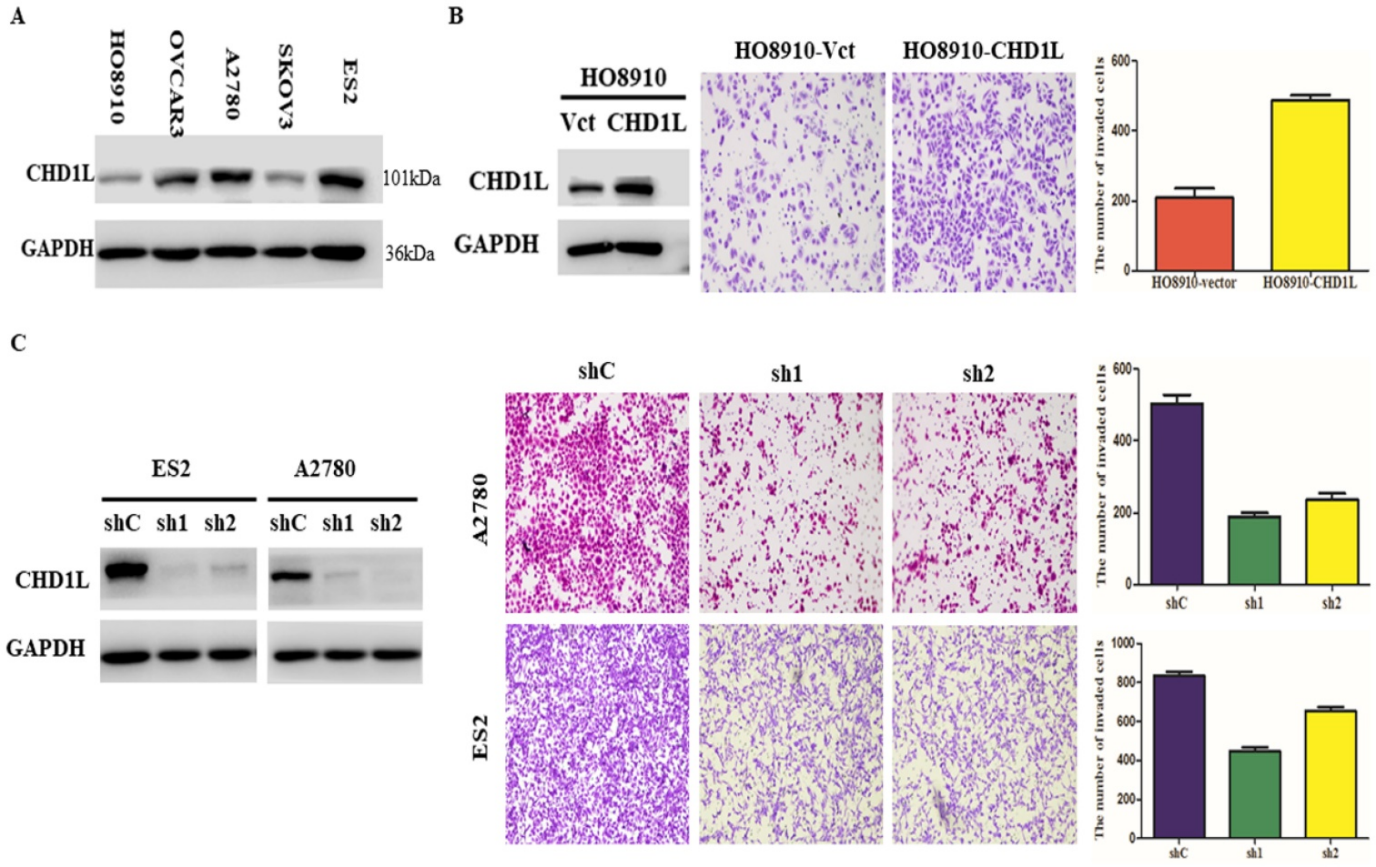
** Overexpression of CHD1L enhances EOC cell invasion and silencing of CHD1L by RNA interference inhibits EOC cell invasion * in vitro*. A.** The expression levels of CHD1L in 5 EOC cell lines by Western blotting analysis. **B.** Overexpression of CHD1L enhances EOC cell invasion * in vitro*. Left: Western blotting reveals that ectopic expression of CHD1L was substantially increased in HO8910-CHD1L cells compared with that in HO8910-vector cells. Right: Ectopic overexpression of CHD1L enhanced HO8910 cell invasion in a Transwell assay. The numbers of invaded cells in the HO8910-CHD1L and control groups are shown in the right panel. Data are the mean ± standard error (SE) of three independent experiments; P<0.05 by Student's t test. **C.** Silencing of CHD1L by RNA interference inhibits EOC cell invasion * in vitro.* Left: Western blotting reveals that CHD1L was efficiently knocked down by the treatment with CHD1L-shRNA1 and CHD1L-shRNA2. Right: Cell invasion was evaluated using a Matrigel invasion chamber. Silencing of CHD1L decreased ES2 and A2780 cells invasive capacity. The numbers of invaded cells in the shCHD1L and control groups are shown in the right panel. Data are the mean ± standard error (SE) of three independent experiments; P<0.05 versus cells transfected with shC by Student's *t*-test.

Based on the five EOC cell lines analyzed, we found that HO8910 cells expressed relatively low levels of endogenous CHD1L protein, and on the contrary, ES2 and A2780 cells expressed relatively high levels of endogenous CHD1L protein (**Fig. 2A**). To evaluate the tumorigenic ability of CHD1L overexpression, HO8910-CHD1L cell lines that were transfected with plasmid [pcDNA3.1(+)-CHD1L] were used to perform an * in vitro* Matrigel invasion assay (**Fig. 2B, left**). We found that the invasive ability of the HO8910-CHD1L cells was significantly stronger than that of the control HO8910-vector cells in the Matrigel invasion assays (*P*<0.05, **Fig. 2B, right**).

### Short hairpin RNA-mediated CHD1L silencing inhibits EOC cell invasion and metastasis *in vitro*

To investigate the effects of CHD1L-silencing on invasion and metastasis, A2780 and ES2 cells were transfected with plasmids for either control shRNA or CHD1L-specific shRNAs that displayed the strongest effect in knocking down endogenous CHD1L expression in EOC cells (**Fig. 2C, left**). Silencing endogenous CHD1L expression by shRNA in A2780 and ES2 cells completely abolished the invasive ability of both the shCHD1L-A2780 and shCHD1L-ES2 cells in Matrigel invasion assays (*P*<0.05, **Fig. 2C, right**).

### CHD1L up‑regulates METAP2 expression in EOC cells

**Figure 3:**
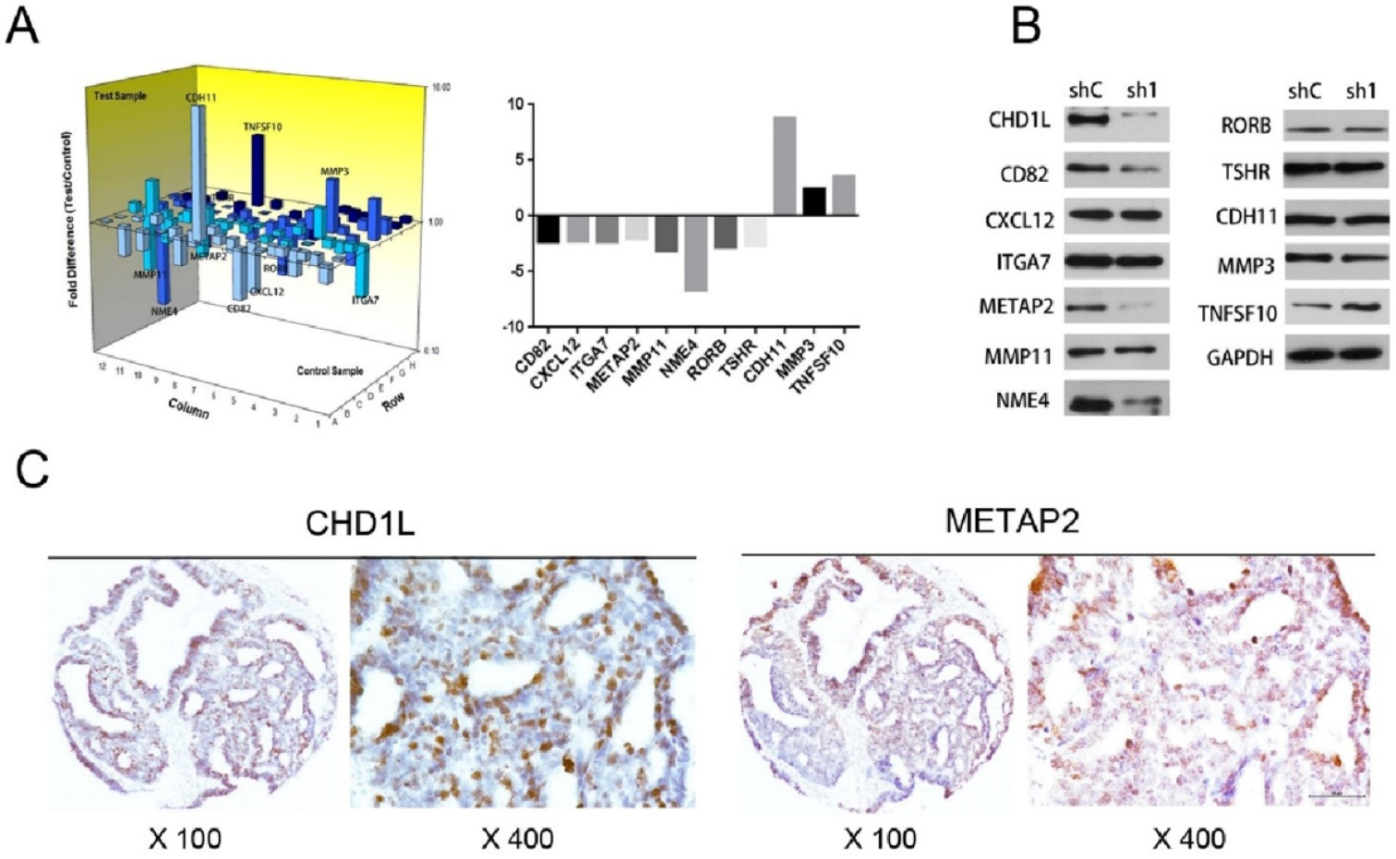
** The associations of CHD1L and METAP2 expression in EOC cells. A.** A total of 11 genes were differentially expressed by more than 2-fold in ES2-shCHD1L-1 cells compared with that in ES2-shControl cells. Eight genes were found to be downregulated (CD82, METAP2, NME4, RORB, CXCL12, TSHR, MMP11 and ITGA7) and another three genes were found to be upregulated (TNFSF10, CDH11 and MMP3), as detected using the Human Tumor Metastasis RT² Profiler™ PCR Array (Super Array Bioscience, America). **B.** Silencing of CHD1L by shRNA down-regulated CD82, METAP2 and NME4 expression in ES2-shCHD1L-1 cells, while up-regulated TNFSF10 expression in ES2-shCHD1L-1 cell, as detected by Western blotting. **C.** The associations of CHD1L and METAP2 expression in EOC patients. Overexpression of CHD1L and high-level expression of METAP2 were detected by IHC in an ovarian carcinoma (case 140), in which more than 80% of carcinoma cells showed strong positive staining of these proteins.

To investigate any underlying downstream genes regulated by CHD1L expression, we extracted RNA of ES2-shControl and ES2-shCHD1L-1 cells, and detected the mRNA expression profiles using the Human Tumor Metastasis RT² Profiler™ PCR Array (Super Array Bioscience, America), which contains 84 cell invasion/metastasis-related genes. Examining the differential expression of these genes revealed that a total of 11 genes were differentially expressed by more than 2-fold in ES2-shCHD1L-1 cells. Eight genes were found to be downregulated (CD82, METAP2, NME4, RORB, CXCL12, TSHR, MMP11 and ITGA7) and three were found to be upregulated (TNFSF10, CDH11 and MMP3) (**Fig. 3A**). Next, we applied Western Blotting to analyzed protein expression levels of these genes. Consistent with the mRNA expression in the real-time PCR array, downregulated protein expression of CD82, METAP2 and NME4 was found in ES2-shCHD1L-1 cells, while upregulated protein expression of TNFSF10 was found in ES2-shCHD1L-1 cells (**Fig. 3B**).

### Correlation between the expression of METAP2 and that of CHD1L in EOC

In order to further confirm whether CD82, METAP2, NME4 and TNFSF10 are potential downstream target genes regulated by CHD1L, we quantified the correlations between the expression levels of these genes and that of CHD1L by IHC in our cohort of EOC tissue microarrays. In 153 of the 160 tissue samples, CHD1L and METAP2 were examined successfully and simultaneously by IHC (**Fig. 3C**). Our results show that the expression levels of CHD1L are significantly associated with METAP2 (*P*<0.05, **Table 3**). Interestingly, we found no significant correlation between the expression levels of CHD1L and CD82, NME4 or TNFSF10.

## Discussion

Although the treatment of EOC has been improved greatly in recent years, the mortality rate is still very high 1. The lack of any good biological markers for early detection or an accurate prognostic prediction is partly responsible for the high mortality of EOC. Our previous study demonstrated the presence of CHD1L overexpression in EOC and reported that overexpression of CHD1L protein is significantly correlated with the metastasis proceeding of ovarian carcinoma. We also reported that CHD1L protein expression, as examined by IHC, may act as a novel prognostic biomarker for patients with EOC 16. In the present study, we increased our sample size and further confirmed that the degree of CHD1L overexpression increased sequentially from the normal ovarian tissues, to ovarian cystadenoma, to borderline tumors, and finally to EOC tissues. Furthermore, CHD1L overexpression is significantly associated with histological type and advanced pT/pN/pM status, as well as FIGO stage of EOC. Similar results have also been observed in human non-small-cell lung cancer, in which overexpression of CHD1L was reported to be associated with lymph node metastasis and/or distant organ metastasis 12. These studies support our data showing that CHD1L plays an important role in the promotion of EOC cell invasion and metastasis.

To date, it is still not clear what the function of CHD1L is. In the present study, we evaluated CHD1L protein expression levels in six different human ovarian carcinoma cell lines (i.e. ES2, OVCAR-3, A2780, HO-8910 and SKOV3) using western blotting analysis. We found that HO8910 cell lines displayed relatively low levels of endogenous CHD1L protein expression, while on the contrary, the ES2 and A2780 cell lines displayed relatively high levels of endogenous CHD1L protein expression. Decreasing expression levels of CHD1L protein was accomplished using a specific lentiviral shRNA, and shRNA-mediated knockdown of endogenous CHD1L positively abolished the invasiveness and/or metastasis of A2780 and ES2 cells. Furthermore, ectogenic overexpression of CHD1L, transfected with plasmid, led to cell invasion and metastasis *in vitro*. These data provide evidence that CHD1L is involved in ovarian carcinoma cell invasion and/or metastasis and that overexpression of CHD1L may be a critical factor in promoting EOC cell metastasis.

However, despite there being a clear connection between CHD1L and cell invasion/metastasis, the precise molecular mechanisms by which CHD1L promotes cancer cell invasion/metastasis are still unclear. A previous study demonstrated that CHD1L upregulates the expression of ARHGEF9, which subsequently activates Cdc42, causing filopodia formation, epithelial-mesenchymal transition (EMT), and finally promotes hepatocellular carcinoma cell invasion and metastasis 19. Li et al. 20 recently demonstrated that CHD1L activates expression of SPOCK1, which activates Akt signaling to then block apoptosis and promote hepatocellular carcinoma cell invasiveness and metastasis in mice. However, little is known about the underlying mechanisms involved in CHD1L-regulated EOC cell invasion and metastasis. To further investigate the downstream molecular events involving CHD1L-regulated invasiveness and metastasis in EOC, we compared mRNA expression profiles between ES2-shCHD1L-1 cells and ES2-shControl cells using a Human Tumor Metastasis real-time PCR array, which contains 84 well-known cell invasion/metastasis-related genes. Of the 84 genes, we identified a total of 11 genes that were differentially expressed by more than 2-fold, including eight genes that were downregulated (CD82, METAP2, NME4, RORB, CXCL12, TSHR, MMP11 and ITGA7) and three genes that were upregulated (TNFSF10, CDH11 and MMP3). Next, protein expression of these differentially regulated genes in the ES2-shCHD1L-1 cells was quantified via western blot assay. Consistent with the mRNA expression data, we found that CD82, METAP2 and NME4 were downregulated and TNFSF10 was upregulated. To further confirm these results, the expression status of these genes (CD82, METAP2, NME4 and TNFSF10) was detected using IHC in our TMA assay. Further correlation analysis revealed that there was no significant correlation between the expression levels of CHD1L and that of CD82, NME4 and TNFSF10, regardless of the cut off value used. Interestingly, we did observe a significant positive correlation between overexpression of CHD1L and METAP2 expression levels. Given this, it is reasonable to propose that CHD1L promotes cell invasion via the regulation of METAP2 expression in our EOC cells.

METAP2 (methionyl aminopeptidase 2) is a glycoprotein that is responsible for the processing of the N-terminal initiator methionine from nascent proteins in cells 21. Upregulated expression of METAP2 promotes cell proliferation 22, while silencing METAP2 using siRNA induces significant inhibition of the proliferation of human umbilical vein endothelial cells 23,24. It has been reported that overexpression of METAP2 is observed in various samples of human malignancies, such as breast cancer, colon cancer, lung cancer, ovarian cancer and prostate carcinomas and promotes the development of tumors 25. A recent study demonstrated that regarding non-small-cell lung cancer (NSCLC), that METAP2 is involved in regulating cell proliferation and apoptosis in NSCLC. Furthermore, METAP2 inhibition may provide prevention of tumor cell growth, development and progression in NSCLC patients 26. However, there is no evidence indicating METAP2 involvement being directly correlated with metastatic potential in cancer, but it has been demonstrated that METAP2 is a new target for the metastasis-associated protein, S100A4 27. Taken in the context of our results here on the function of CHD1L in EOC cells, these observations indicate that METAP2 might be a potential downstream target in the aggressive behavior of CHD1L-mediated EOC, and as a result promotes cancer cell invasion and metastasis. Further studies are obviously needed to elucidate the precise molecular mechanisms of CHD1L regulation of METAP2 in EOC. Furthermore, it may lead to a more effective management of EOC progression, via precise prognostication and treatment targeted at these molecules.

Here based on our previous study and the present study, we show that high expression of CHD1L plays a potential role in the inducement of EOC cancer cell invasion and/or metastasis via the regulation of METAP2 expression. Furthermore we showed that silencing CHD1L, through inhibition of CHD1L expression levels may be a promising therapeutic strategy for prevention and intervention of metastasis of EOC.

## Materials and Methods

### Patients and tissue microarray

In this study, paraffin-embedded pathological specimens from 160 patients diagnosed with EOC and treated between 2003 and 2010 were obtained from the archives of the Department of Pathology, the First Affiliated Hospital, Sun Yat-Sen University, Guangzhou, China, and prepared for ovarian tissue microarray (TMA). The patients were selected based on availability of resection tissue, as well as follow-up data. Any patients who had accepted preoperative radiation or chemotherapy were excluded from the study. In addition, patients that died from unknown causes or emergency were also excluded from this study. The TMA was constructed according to a method described previously 17. Prior patient's informed consent and the Institute Research Medical Ethics Committee of Sun Yat-Sen University granted approval were obtained for the research purposes of this study.

### Immunohistochemistry staining

The immunohistochemical studies were carried out using a standard streptavidin-biotin-peroxidase complex method demonstrated previously 18. Briefly, tissue sections were deparaffinized and rehydrated. The endogenous peroxidase activity of the tissue was blocked using 3% hydrogen peroxide for 10 min. For antigen retrieval, slides were immersed and boiled in 10 mM citrate buffer (pH 6.0) for 15 min in a pressure cooker, and then non-specific binding was blocked by 5% normal goat serum for 10 min. The slides were incubated with anti-CHD1L (1:100 dilution; Abcam), a 1:200 dilution of monoclonal antibody against cluster of differentiation 82 (CD82), non-metastatic cells 4 (NME4), tumor necrosis factor (ligand) superfamily member 10 (TNFSF10) or methionyl aminopeptidase 2 (METAP2) at 4°C overnight in a moist chamber. The slides were sequentially incubated with biotinylated goat anti-mouse IgG (Santa Cruz Biotechnology) at a dilution of 1:100 and then allowed to react with the streptavidin-peroxidase conjugate, each for 30 min at room temperature. Staining was developed using the 3, 5-diaminobenzidine (DAB) Substrate Kit (Dako) according to the manufacturer's instructions, followed by Mayer hematoxylin counterstaining.

### EOC cell lines and culture conditions

The EOC cell lines ES2, HO8910 and SKOV3 were maintained in RPMI-1640 medium, and the EOC cell lines A2780 and OVCAR3 were cultured in DMEM medium, both of which supplemented with 10% fetal bovine serum (FBS) (GIBCO) and 1% antibiotics (100 U/mL penicillin and 100 μg/mL streptomycin).

### Protein extraction and Western blotting

Protein was extracted from the EOC cells by Radio-Immunoprecipitation Assay (RIPA) Lysis Buffer (Beyotime) at 4 °C. Protein concentrations were detected using the Bicinchoninic Acid (BCA) Protein Assay Kit (BioRad, Hercules, CA, USA). Equal amounts of whole-cell lysates were separated by SDS-polyacrylamide gel electrophoresis and transferred onto a polyvinylidene difluoride (PVDF) membrane (Millipore, Danvers, MA, USA) and then incubated with primary mouse monoclonal antibodies against the following human proteins: CHD1L (1:1000 dilution), METAP2 (1:1000 dilution), TNFSF10 (1:1000 dilution), CD82 (1:1000 dilution), and NME4 (1:1000 dilution) (BD Transduction Laboratories) overnight at 4 °C. GAPDH was used as a loading control (1:5000 dilution, BD Transduction Laboratories). After the PVDF membranes were incubated with the secondary antibody (goat anti-mouse, 1:10,000 dilution, Cell Signaling Technology, Danvers, MA, USA), the immunoreactive proteins were visualized by enhanced chemiluminescence detection reagents (Amersham Biosciences, Uppsala, Sweden) in accordance with the manufacturer's instructions.

### Plasmid Constructs and Transfection

To evaluate the tumorigenic ability of CHD1L, the full-length CHD1L cDNA was amplified by PCR and cloned into a pcDNA3.1+ expression vector (Invitrogen) as described previously 8. The expression plasmids were transfected into HO9810 cells using Lipofectamine 2000 (Invitrogen) according to the manufacturer's instructions. Stable CHD1L expressing clones were selected using Geneticin (Roche), and the level of CHD1L expression was detected by Western blot analysis. Cells transfected with empty vector were used as controls.

### Knockdown of CHD1L by lentiviral short hairpin RNA (shRNA)

Based on the CHD1L sequence, we constructed a specific lentiviral shRNA for CHD1L using the sequences of shRNA1 (5′-GCC AAG AGA AGG AGA-3′) and shRNA2 (5′-CGT ATT GGA CAT GCC ACG AAA-3′), which have been previously verified to efficiently knockdown endogenous CHD1L expression in human cancer cells 19. The lentiviral particles were collected, filtered, and reserved 72 h post transfection. Next, we transfected the EOC cell lines with the lentivirus particles, each transfection was performed in triplicate and repeated three times. Cells were harvested 72 h post transfection and knockdown efficiency was quantified using Western blotting.

### Cell invasion assay

Post-second infection, cells were seeded onto a synthetic basement membrane (Falcon TM Cell Culture Inserts, BD, Oxford) located at the bottom of the wells in a 24-well culture plate. In our invasion assay, polycarbonate filters (8 μm pore size) were coated with 20 μg Matrigel and placed in a modified Boyden chamber. Fetal bovine serum was added to the lower chamber as a chemoattractant. Cells were then incubated at 37°C and allowed to invade through the Matrigel barrier for either 18 or 36 h. After the incubation period, non-invading cells on the upper filter were cleaned up by a cotton swab and invading cells on the bottom of the filter were fixed and stained with crystal violet. The invasive cells were then quantified in five fields under an inverted microscope. Experiments were performed in triplicate with a minimum of 40 grids (×400 magnification) per filter counted.

### Real-time polymerase chain reaction gene array

Post the second dose of shRNA knockdown experiments, we extracted RNA from shCHD1L and shC using Trizol (Invitrogen) and cleared them by RNeasy® Min Elute^TM^ Cleanup Kit (Qiagen, Valencia, CA). Total RNA was reverse transcribed through Super-Script III Reverse Transcriptase (Invitrogen) and polymerase chain reaction (PCR) was used to amplify complementary DNA using the 2× Super Array PCR master mix (Super Array Bioscience, Frederick, MD). An Opticon^TM^ DNA Engine ABI PRISM7900 system (Applied Biosystems, Foster City, CA) was used for Real-time PCR on each sample using the Human Tumor Metastasis RT^2^ Profiler^TM^ PCR Array (Super Array Bioscience), following the manufacturer's instructions. Finally, data was normalized to GAPDH levels using the ΔΔCt method.

### Statistical analyses

The SPSS statistical software package (SPSS Standard version 20.0, SPSS Inc. Chicago, IL) was used for statistical analysis. Data are presented as the mean ± standard deviation (SD) of 3 independent experiments. The independent Student's t test was performed to compare the invasive ability between any 2 pre-selected groups. The association of CHD1L expression with clinicopathologic features of EOC patients and the correlation between CHD1L expression and METAP2 expression in the EOC clinical samples were evaluated using the χ^2^ test. Survival curves were obtained through the Kaplan-Meier method. Log-rank test was used to compare different survival curves. P values less than 0.05 were considered to be statistically significant.

## Figures and Tables

**Table 1:** The expression of CHD1L in normal ovaries and in benign and malignant epithelial ovarian tumors^a^

	All cases	CHD1L protein	
		Normal expression	Overexpression
Normal ovaries	30	30 (100%)	0 (0)
Cystadenomas	35	33 (94%)	2 (6%)
Borderline tumors	40	35 (87%)	5 (13%)
Invasive carcinomas	160	75 (47%)	85 (53%)

^a^ Values are n (%). A significant increasing frequency of intensive expression of CHD1L was observed in cystadenomas, in borderline tumors and in invasive carcinomas (*P*<0.01, Chi-Square Test for Trend).

**Table 2:** Association of CHD1L expression with patient's clinico-pathological features in 160 ovarian carcinomas

	All cases	CHD1L protein		
		Normal expression	Overexpression	*P* value ^a^
**Age at surgery (years)**				0.620
<51.0 ^b^	82	40 (49%)	42 (51%)	
≥51.0	78	35 (45%)	43 (55%)	
**Histological type**				0.024
Serous	106	43 (41%)	63 (59%)	
Mucinous	19	14 (74%)	5 (26%)	
Others^c^	35	18 (51%)	17 (49%)	
**Histological grade (Silveberg)**				0.142
G1	28	16 (57%)	12 (43%)	
G2	89	44 (49%)	45 (51%)	
G3	43	15 (35%)	28 (65%)	
**pT status**				<0.001
pT1	38	29 (76%)	9 (24%)	
pT2	35	15 (43%)	20 (57%)	
pT3	87	31 (36%)	56 (64%)	
**pN status**				<0.001
pN0	77	50 (65%)	27 (35%)	
pN1	83	25 (30%)	58 (70%)	
**pM status**				0.013
pMX	135	69 (51%)	66 (49%)	
pM1	25	6 (24%)	19 (76%)	
**FIGO stage**				<0.001
I	25	20 (92%)	5 (20%)	
II	21	13 (52%)	8 (38%)	
III	89	36 (40%)	53 (60%)	
IV	25	6 (24%)	19 (76%)	

^a^ Chi-square test; ^b^ Mean age; ^c^ Endometrioid, Clear cell and Undifferentiated types.

**Table 3:** Association of CHD1L expression with METAP2 in 153 EOC

Variable	All cases	CHD1L protein		
		Normal expression	Overexpression	*P* value ^a^
METAP2				0.020
Low-level expression	74	42 (57%)	32 (43%)	
High-level expression	79	30 (38%)	49 (62%)	

^a^ Chi-square test.
